# Abdominal versus perineal approach for external rectal prolapse: systematic review with meta-analysis

**DOI:** 10.1093/bjsopen/zrac018

**Published:** 2022-04-07

**Authors:** Gianluca Pellino, Giacomo Fuschillo, Costantinos Simillis, Lucio Selvaggi, Giuseppe Signoriello, Danilo Vinci, Christos Kontovounisios, Francesco Selvaggi, Guido Sciaudone

**Affiliations:** Colorectal Surgery, Department of Advanced Medical and Surgical Sciences, Università degli Studi della Campania ‘Luigi Vanvitelli’, Naples, Italy; Colorectal Surgery, Vall d’Hebron University Hospital, Barcelona, Spain; Colorectal Surgery, Department of Advanced Medical and Surgical Sciences, Università degli Studi della Campania ‘Luigi Vanvitelli’, Naples, Italy; Cambridge Colorectal Unit, Addenbrookes Hospital, Cambridge University Hospitals NHS Foundation Trust, Cambridge, UK; Colorectal Surgery, Department of Advanced Medical and Surgical Sciences, Università degli Studi della Campania ‘Luigi Vanvitelli’, Naples, Italy; Section of Statistic, Department of Experimental Medicine, Università degli Studi della Campania ‘Luigi Vanvitelli’, Naples, Italy; Colorectal Surgery, Department of Advanced Medical and Surgical Sciences, Università degli Studi della Campania ‘Luigi Vanvitelli’, Naples, Italy; Department of Colorectal Surgery, Royal Marsden Hospital, London, UK; Department of Colorectal Surgery, Chelsea and Westminster Hospital, London, UK; Department of Surgery and Cancer, Imperial College London, London, UK; Colorectal Surgery, Department of Advanced Medical and Surgical Sciences, Università degli Studi della Campania ‘Luigi Vanvitelli’, Naples, Italy; Colorectal Surgery, Department of Advanced Medical and Surgical Sciences, Università degli Studi della Campania ‘Luigi Vanvitelli’, Naples, Italy

## Abstract

**Background:**

External rectal prolapse (ERP) is a debilitating condition in which surgery plays an important role. The aim of this study was to evaluate the outcomes of abdominal approaches (AA) and perineal approaches (PA) to ERP.

**Methods:**

This was a PRISMA-compliant systematic review with meta-analysis. Studies published between 1990 and 2021 were retrieved. The primary endpoint was recurrence at the last available follow-up. Secondary endpoints included factors associated with recurrence and function. All studies were assessed for bias using the Newcastle–Ottawa Scale and Cochrane tool.

**Results:**

Fifteen studies involving 1611 patients (AA = 817; PA = 794) treated for ERP were included, three of which were randomized controlled trials (RCTs; 114 patients (AA = 54; PA = 60)). Duration of follow-up ranged from 12 to 82 months. Recurrence in non-randomized studies was 7.7 per cent in AA *versus* 20.1 per cent in PA (odds ratio (OR) 0.29, 95 per cent confidence interval (c.i.) 0.17 to 0.50; *P* < 0.001, *I^2^* = 45 per cent). In RCTs, there was no significant difference (9.8 per cent *versus* 16.3 per cent, AA *versus* PA (OR 0.82, 95 per cent c.i. 0.29 to 2.37; *P* = 0.72, *I^2^* = 0.0 per cent)). Age at surgery and duration of follow-up were risk factors for recurrence. Following AA, the recurrence rates were 10.1 per cent and 6.2 per cent in patients aged 65 years and older and less than 65 years of age, respectively (effect size [e.s.] 7.7, 95 per cent c.i. 4.5 to 11.5). Following PA, rates were 27 per cent and 16.3 per cent (e.s. 20.1, 95 per cent c.i. 13 to 28.2). Extending follow-up to at least 40 months increased the likelihood of recurrence. The median duration of hospital stay was 4.9 days after PA *versus* 7.2 days after AA. Overall, incontinence was less likely after AA (OR 0.32), but constipation occurred more frequently (OR 1.68). Most studies were retrospective, and several outcomes from RCTs were not consistent with those observed in non-RCTs.

**Conclusion:**

The overall risk of recurrence of ERP appears to be higher with PA *versus* AA. Incontinence is less frequent after AA but at the cost of increased constipation. Age at surgery and duration of follow-up are associated with increased risk of recurrence, which warrants adequate reporting of future studies on this topic.

## Teaser

Recurrence after external rectal prolapse appears to be higher after perineal *versus* abdominal repair; however, the findings of randomized controlled trials do not confirm such a finding. Duration of follow-up and age at surgery are prognosticators of recurrence. Some functional issues can persist or appear after surgery. Quality of life has been poorly reported and investigated in this population of patients.

## Introduction

External rectal prolapse (ERP) is defined as circumferential, full-thickness prolapse of the rectal wall that protrudes outside the anal canal^1,2^. ERP is also referred to as rectal procidentia or ‘complete’ prolapse, and is often associated with incontinence or defaecation disorders, resulting in a state of significant discomfort and impaired quality of life.

The incidence of ERP has been reported to be six times higher in women, especially those over the age of 60 years^3^. Initial management is usually conservative or used as a temporizing measure prior to the consideration of surgery. The latter is the only ‘curative’ approach to reduce the ERP if it is irreducible, and it may improve some of the accompanying symptoms. Several surgical techniques have been proposed, which can be divided into perineal approaches (PA) and abdominal approaches (AA). The most common PA procedures are the Delorme procedure and the Altemeier procedure (perineal recto-sigmoidectomy)^4^. Rectopexy and resection rectopexy (with or without mesh placement) are the most performed AA procedures. Historically, PA has been offered to older patients, owing to the lower morbidity associated with the procedure. However, recent advances in perioperative management, and laparoscopic and robotic surgery allow AA to be performed safely in selected older, fit patients^4^.

Although both approaches are feasible^2^, the recurrence rates are presumed to be higher with PA, reported to be as high as 14 to 27 per cent^5^. However, the superiority of AA over PA has been questioned by recent randomized controlled trials (RCTs)^6,7^. PA might be associated with fewer complications, justifying its use in frail patients. In addition, the functional outcome of patients after the restoration of anatomy, and whether this can be influenced by surgical approach is unclear.

The aim of this systematic review was to assess the risk of recurrence in patients treated with PA *versus* AA for ERP, focusing on factors associated with increased risk. Secondary aims included the impact of surgical approach on postoperative function and health-related quality of life (HRQoL).

## Methods

The systematic review with meta-analysis was performed following the PRISMA statement^8^ and Meta-analysis of Observational Studies in Epidemiology (MOOSE) checklist^9^. The trial was registered in PROSPERO (CRD42020176890).

### Search strategy and data sources

A literature search was conducted in MEDLINE (PubMed) and Embase for articles published between 1990 and 2021. Two authors independently performed the literature screening (Gu.S. and G.F.). The following data were independently extracted from the included studies: first author, journal, year of publication, study type, number of patients (AA and PA), mean patient age, and median duration of follow-up. The search terms used were a combination of ‘Delorme’ or ‘Altemeier’ or ‘Rectopexy’ or ‘perineal approach’ or ‘abdominal approach’ AND ‘rectal prolapse’. The full search details are available in *Table S1*. A cross-reference search was done, searching the reference list of included articles.

### Inclusion and exclusion criteria

Studies comparing the outcomes of ERP treated by AA *versus* PA were evaluated for inclusion. For studies originating from the same centre, only the most recent study with the more complete data was included. Non-comparative studies, studies with calculable endpoints with fewer than 20 patients, studies with a follow-up of less than 12 months, and those published before 1990 were excluded.

### Endpoints

The primary endpoint was recurrence at the last available follow-up, defined as the presence of full-thickness prolapse after surgery as diagnosed by rectal examination.

The secondary endpoints were factors associated with the risk of recurrence, including age at surgery and duration of follow-up; duration of hospital stay; postoperative constipation or incontinence; and HRQoL.

### Statistical analysis

The meta-analysis was performed according to the Cochrane Collaboration the Quality of Reporting of Meta-analyses (QUORUM)^9^ guidelines. The estimated effect measures are reported as odds ratios (OR) with 95 per cent confidence intervals (c.i.). The ratio represented the probability of occurrence of an adverse event in the group of patients operated on with AA *versus* the group of patients operated on with PA. An OR of less than 1 indicated worse outcomes of PA, and the point estimate of OR was considered statistically significant if the 95 per cent c.i. did not include the value ‘1’. For studies that included a zero in a cell for the number of events, the Haldane correction was applied, adding a value of 0.5 in both groups from the respective study^10^. With regard to follow-up, the cut-off value was chosen using the longest available follow-up allowing for even distribution of the included studies. Odds ratios were combined with the Mantel–Haenszel χ^2^ method using the random-effect technique^11^.

### Risk-of-bias assessment

RCTs and non-randomized trials were analysed separately. All studies were graded using the Newcastle–Ottawa Scale^12^. The risk of bias in selected studies was performed using the Cochrane tool risk of bias 2.0^13^ for RCTs and non-RCTs, and the ROBINS-I tool for non-RCTs^14^.

The publication bias for analysis including at least 10 studies was assessed by means of funnel plot inspection (*Fig. S5*).

The overall strength of evidence was assessed with the GRADE approach^15^.

## Results

**Fig. 1:**
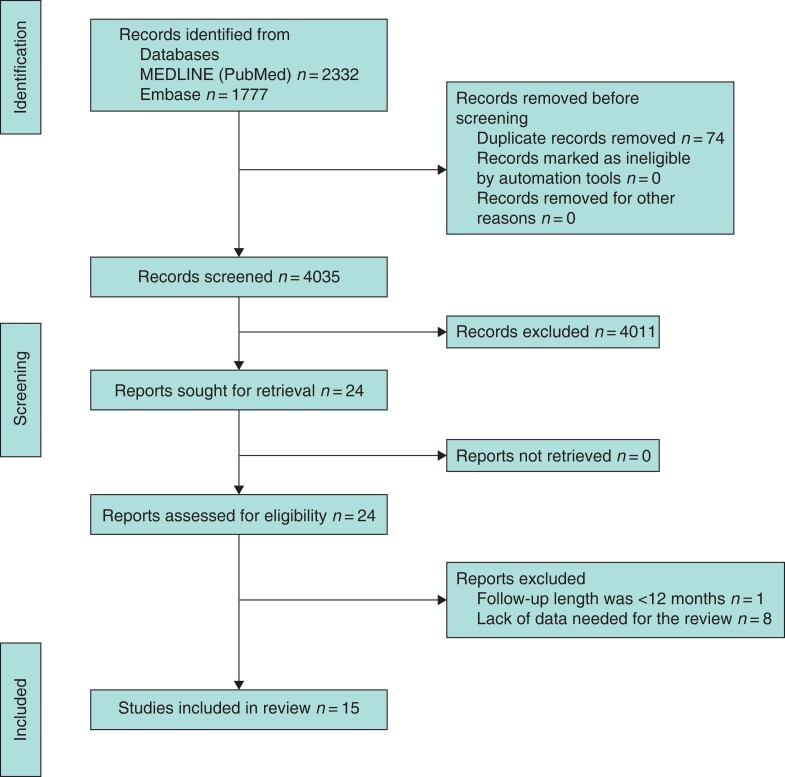
Flowchart of study selection for the current meta-analysis according to PRISMA statement

The search yielded 4058 studies, which were analysed by titles and abstracts; 74 duplicates were excluded. All non-comparative studies, reviews, meta-analyses, and case reports were excluded. Twenty-four full-text articles were reviewed, with eight being excluded owing to lack of data needed for the review and one because the duration of follow-up was less than 12 months. Fifteen comparative studies met the inclusion criteria^3–7,16–27^. These included 12 retrospective studies^3,17–20,21–27^ and three RCTs^6,7,16^ (*Table 1*). The flowchart for study inclusion is reported in *Fig. 1*. There was 100 per cent agreement among reviewers in the extraction of data, reported in a Microsoft^®^ Excel spreadsheet. In total, 1611 patients who underwent ERP repair between 1990 and 2021 were included in the analysis: 817 patients (50.7 per cent) with AA and 794 patients (49.3 per cent) with PA (*Table 1*). Duration of follow-up ranged from 12 to 82 months. The types of AA used in the different studies were rectopexy with^3,7,16–21,23,27^ or without sigmoid resection^3,7,17–20,22,24,25,27^ (both open and laparoscopic), ventral rectopexy (*D’Hoore*)^6,17,25,26^, and rectopexy with mesh (*Wells*)^3,21,23^. The PA types used were Delorme^3,6,7,17–21,23–25^, perineal rectosigmoidectomy (*Altemeier*)^3,7,16,17,20–27^, and *Thiersh*^19,25^.

**Table 1:** Characteristics of included studies

Author	Year	Study type	No. of patients		Mean age (years)	Mean duration of FU (months)	NOS
			AA	PA			
**Deen *et al.*^16^**	1994	Randomized	10	10	68.5	17	7
**Boccasanta *et al*.^17^**	1999	Retrospective	25	10	60.6	35	4
**Kim *et al*.^18^**	1999	Retrospective	176	183	63.7	72	5
**Aitola *et al*.^19^**	1999	Retrospective	104	8	59	62	4
**Sobrado *et al*.^26^**	2004	Retrospective	36	15	56.7	49	4
**Hammond *et al*.^20^**	2007	Retrospective	13	62	60.8	39	4
**Pescatori and Zbar^21^**	2009	Retrospective	42	75	57	61	5
**Riansuwan *et al*.^3^**	2010	Retrospective	122	55	59.9	43.8	5
**Lee *et al*.^22^**	2011	Retrospective	8	123	80.1	12.4	4
**Senapati *et al*.^7^**	2013	Randomized	19	25	63	36	8
**Lee *et al*.^23^**	2014	Retrospective	64	40	57.7	24.4	5
**Mik *et al*.^24^**	2015	Retrospective	18	68	67	32	6
**Emile *et al*.^6^**	2017	Randomized	25	25	39.7	18	7
**Gleditsch *et al*.^25^**	2018	Retrospective	73	20	72	82	5
**Ng *et al*.^27^**	2019	Retrospective	82	75	73.1	60	5

AA, abdominal approach; PA, perineal approach; FU, follow-up; NOS, Newcastle–Ottawa Scale.

### Postoperative recurrence

**Fig. 2:**
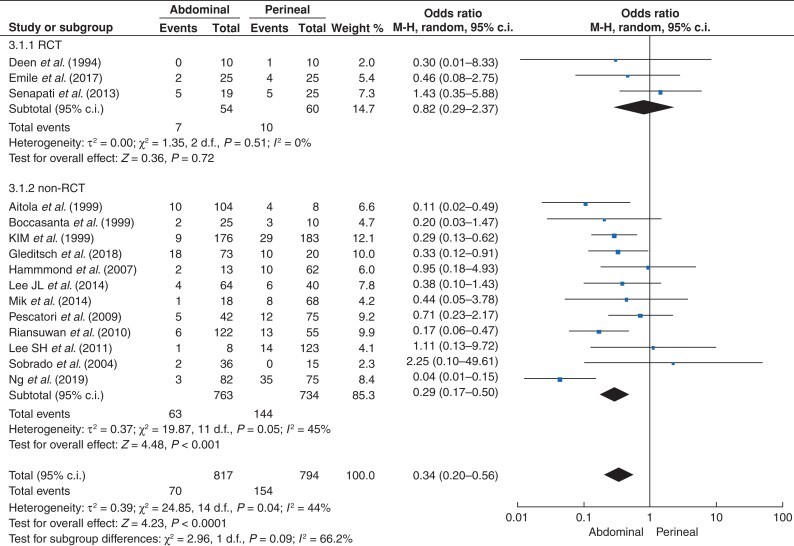
Recurrence with an abdominal approach (AA) *versus* perineal approach (PA) to external rectal prolapse

Non-RCT studies reported a lower risk of recurrence with AA than with PA (OR 0.29, 95 per cent c.i. 0.17 to 0.50; *P* < 0.001, *I^2^* = 45 per cent) (*Fig. 2*). Overall effect size (ES) in non-RCT trials was 7.7 (95 per cent c.i. 4.5 to 11.5) in AA *versus* 20.1 (95 per cent c.i. 13 to 28.2) in PA.

Forest plot with odds ratio of single studies divided into randomized controlled trials (RCTs), non-RCTs, and overall odds ratio. c.i., confidence interval.

In RCTs, there was no difference (*P* = 0.720) in the risk of recurrence with the AA *versus* PA (9.8 per cent *versus* 16.3 per cent (OR 0.82, 95 per cent c.i. 0.29 to 2.37; *P* = 0.720, *I^2^* = 0 per cent)) (*Fig. 2*). The heterogeneity was 0.0 per cent. The three studies reported an OR lower than 1, with the exception of that of Senapati *et al*. (OR 1.43)^7^. The overall effect size of recurrence across RCTs was 9.8 for AA (95 per cent c.i. 0.1 to 27.5), while for PA it was 16.3 (95 per cent c.i. 7.3 to 27.4).

Funnel plot inspection did not require any further assessment for publication bias.

### Factors associated with recurrence

Two groups were established according to patient’s age at surgery: one containing studies in which the average patient age was greater than or equal to 65 years, and another containing studies with patients whose average age was less than 65 years.

In non-RCT, AA had a recurrence rate of 10.1 per cent (95 per cent c.i. 0.7 to 25.9) in studies with a mean patient age of more than 65 years *versus* 6.2 per cent (95 per cent c.i. 4.2 to 8.4) in studies with a mean patient age of less than 65 years (*Fig. S1*). The recurrence rate for PA in studies with patients with a mean age of more than 65 years was 27 per cent (95 per cent c.i. 9.2 to 49.7) *versus* 16.3 (95 per cent c.i. 10.9 to 22.3) in studies with patients with a mean age of less than 65 years (*Fig. S2*).

In RCTs, the recurrence rates in studies on patients aged more than 65 years were 0 in AA *versus* 10 per cent in PA, whereas they were 16 per cent in AA and 18 per cent in PA if the mean patient age was less than 65 years.

A cut-off value of 40 months of follow-up allowed for even distribution of the studies into two categories. Studies were divided into two groups, according to duration of follow-up: one with studies with an average duration of follow-up of more than 40 months, and one with studies with an average duration of follow-up of less than 40 months. This analysis was only conducted on non-RCTs because all RCTs had a mean duration of follow-up of less than 40 months. In non-RCTs, recurrence rates increased with increasing duration of follow-up: 8.3 per cent (95 per cent c.i. 4.2 to 13.4) after AA and 24.9 per cent (95 per cent c.i. 12.9 to 39) after PA in studies with more than 40 months of follow-up; and 6.7 per cent (95 per cent c.i. 2.3 to 12.4) after AA and 12.8 per cent (95 per cent c.i. 9 to 17) after PA in those studies with a duration of follow-up of less than 40 months (*Figs S3 and S4*).

### Postoperative function and duration of postoperative stay

**Fig. 3:**
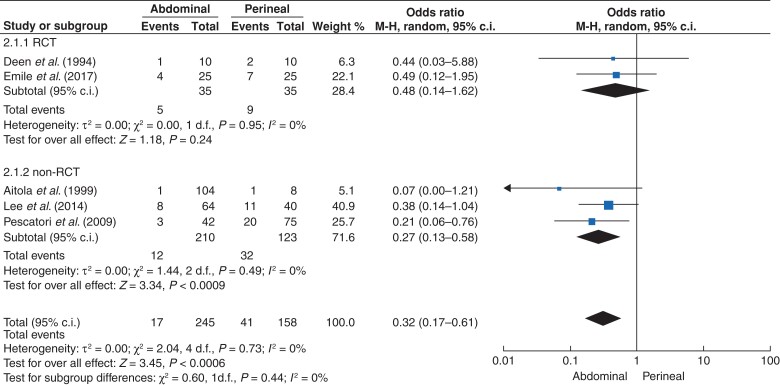
Postoperative faecal incontinence with an abdominal approach (AA) *versus* perineal approach (PA) to external rectal prolapse

**Fig. 4:**
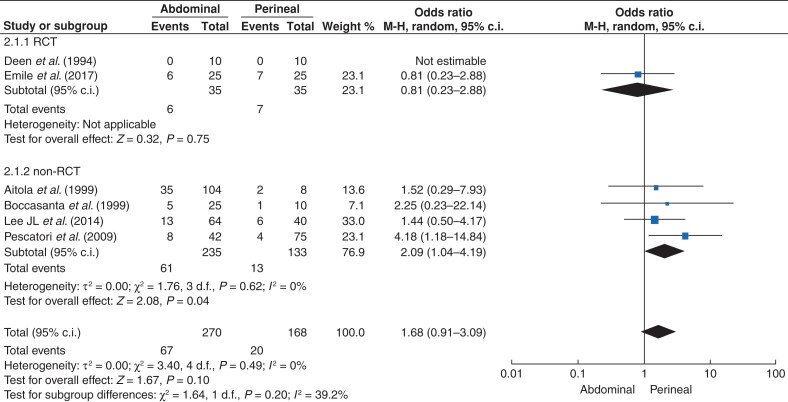
Postoperative constipation with an abdominal approach (AA) *versus* perineal approach (PA) to external rectal prolapse

Data were available on incontinence and postoperative constipation in five studies^6,16,19,21,23^. In RCTs, the number of patients who had postoperative faecal incontinence was five for AA and nine for PA (OR 0.48, 95 per cent c.i. 0.14 to 1.62; *P* = 0.24, *I^2^* = 0) (*Fig. 3***)**. Findings were consistent between RCTs and non-RCTs (*Fig. 3*). Six studies^6,16,17,19,21,23^ provided results for postoperative constipation. In non-RCTs, the OR for constipation was 2.09 (95 per cent c.i. 1.04 to 4.19; *P* = 0.04, *I^2^* = 0) in AA *versus* PA; however, in RCTs, the OR for constipation was 0.81 (95 per cent c.i. 0.23 to 2.88; *P* = 0.75, *I^2^* = 0) (*Fig. 4*).

Forest plot with odds ratio of studies reporting data on faecal incontinence divided into randomized controlled trials (RCTs), non-RCTs, and overall odds ratio. c.i., confidence interval.

Forest plot with odds ratio of studies reporting data on constipation divided into randomized controlled trials (RCTs), non-RCTs, and overall odds ratio. c.i., confidence interval.

Eight of 15 studies provided data on duration of postoperative stay^3,6,16,18,20,22,23,27^. Median duration of postoperative stay was 4.9 days for PA and 7.2 days for AA.

### Health-related quality of life

Only three studies^3,6,7^ reported on HRQoL outcomes following surgery for ERP.

Riansuwan *et al*.^3^ assessed HRQoL using the 36-Item Short Form Survey (SF-36) score, reporting lower mean scores in all domains in the PA with a statistically significant difference in both physical and mental components. In contrast, Emile *et al*.^6^, using Faecal Incontinence Quality of Life Questionnaire (FIQL) and Gastrointestinal Quality of Life Index (GIQIL) scores, did not find any difference between the two groups.

According to Senapati *et al*.^7^, there was no difference between AA and PA using the EuroQol-5 Dimension (EQ-5D) score.

### Risk of bias

**Fig. 5:**
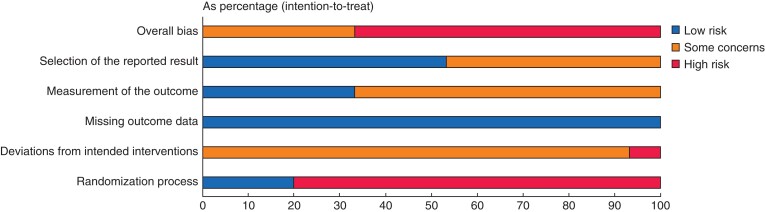
Cochrane risk-of-bias tool outcomes for studies included in the review

A summary of the risk of bias is depicted in *Fig. 5* and *Table 2*. The overall bias was high in 65 per cent of the studies and medium among the remaining 35 per cent, due to the higher percentage of selected non-randomized studies, which is reflected in the randomization process.

**Table 2:** ROBIN I tool: risk of bias in non-randomized controlled trials

Study (year)	Bias due to confounding	Bias in selection of participants into the study	Bias in classification of intervention	Bias due to deviations from intended interventions	Bias due to missing data	Bias in measurement of outcomes	Bias in selection of the reported results	Overall bias
**Ng *et al.* (2019)**	LOW	MODERATE	LOW	LOW	LOW	LOW	MODERATE	LOW
**Gleditsch *et al*. (2018)**	LOW	MODERATE	LOW	LOW	LOW	LOW	LOW	LOW
**Mik *et al.* (2014)**	LOW	MODERATE	LOW	LOW	MODERATE	LOW	MODERATE	LOW
**Pescatori *et al*. (2009)**	LOW	MODERATE	LOW	LOW	LOW	LOW	LOW	LOW
**Riansuwan *et al*. (2010)**	LOW	MODERATE	LOW	LOW	LOW	LOW	MODERATE	LOW
**Lee *et al*. (2011)**	LOW	MODERATE	LOW	LOW	LOW	LOW	MODERATE	LOW
**Lee *et al*. (2014)**	LOW	SERIOUS	LOW	LOW	MODERATE	LOW	MODERATE	MODERATE
**Boccasanta *et al*. (1999)**	LOW	MODERATE	LOW	LOW	LOW	LOW	LOW	LOW
**Hammond *et al*. (2007)**	LOW	SERIOUS	LOW	LOW	MODERATE	LOW	MODERATE	MODERATE
**Kim *et al*. (1999)**	LOW	MODERATE	LOW	LOW	LOW	LOW	LOW	LOW
**Aitola *et al*. (1999)**	LOW	MODERATE	LOW	LOW	LOW	LOW	MODERATE	LOW
**Sobrado *et al*. (2004)**	LOW	SERIOUS	LOW	LOW	MODERATE	LOW	MODERATE	MODERATE

A low risk of bias was observed in the missing outcome data (100 per cent) and in the selection of the reported results (55 per cent). *Table 3* summarizes the quality of evidence among the studies.

**Table 3:** GRADE score: a consensus on rating quality of evidence and strength of recommendations

Outcome		Pooled effect estimates		Pooled relative effects (95% c.i.)	Number of patients/studies	Heterogeneity *I^2^*	*P* for overall effect estimate	Quality of evidence (GRADE)
**Comparison 1: abdominal approach *versus* perineal approach (non-RCTs)**								
		*AA*	*PA*					
Recurrence		0.08	0.2	OR 0.29 (0.17–0.50)	1497/12	0.45	< 0.001	+++
	*Age at surgery (years)*	*Recurrence rate (%) (95% c.i.)*			*No. of studies*			
	> 65	10.1 (0.7–25.9)	27 (9.2–49.7)		4			+
	< 65	6.2 (4.2–8.4)	16.3 (10.9–22.3)		8			++
	*Follow-up duration (months)*							
	> 40	8.3 (4.2–13.4)	24.9 (12.9–39)		7			++
	≤ 40	6.7 (2.3–12.4)	12.8 (9–17)		5			+
Postoperative constipation		0.26	0.1	OR 2.09 (1.04–4.19)	368/4	0	0.04	+++
Postoperative incontinence		0.06	0.26	OR 0.27 (0.13–0.58)	333/3	0	< 0.001	+++
**Comparison 2: AA *versus* PA (RCTs)**								
		*AA*	*PA*					
Recurrence		0.13	0.17	OR 0.82 (0.29–2.37)	114/3	0	0.72	++++
	*Age at surgery (years)*	*Recurrence rate (%)*			*No. of studies*			
	> 65	0	10		4			++
	< 65	16	18		8			+++
	*Follow-up duration (months)*							
	> 40	NA	NA					
	≤ 40	NA	NA					
Postoperative constipation		0.17	0.2	OR 0.81 (0.23–2.88)	013/2	0	0.75	++++
Postoperative incontinence		0.14	0.26	OR 0.48 [0.14–1.62]	014/2	0	0.24	++++

GRADE score: the quality of evidence for most outcomes varied from very low to high quality. All the outcomes that do not include randomized controlled trials (RCTs) were downgraded. c.i., 95% confidence intervals; AA, abdominal approach; PA, perineal approach; OR, odds ratio; NA, not available.

## Discussion

This meta-analysis found that long-term recurrence is higher after PA than after AA for ERP. PA required a shorter postoperative stay. However, this difference was not statistically significant in RCTs. A patient age of less than 65 years at the time of surgery predicted a higher risk of recurrence. The number of recurrences was linked to the duration of FU, being higher for PA in studies with more that 40 months of follow-up, whereas it was higher in studies with shorter follow-up after AA. HRQoL has been poorly investigated, and with different tools. An improvement in overall HRQoL can be expected with both approaches, which is more pronounced in the short term; however, some functional issues might persist or occur after surgery, including a higher likelihood of incontinence after PA *versus* higher rates of constipation after AA.

Although several studies evaluated the long-term results of surgery for ERP, data from meta-analyses comparing the PA with the AA are lacking. This review found that PA tripled the long-term risk of recurrence *versus* AA in studies with long follow-up periods. The PROSPER trial^7^ aimed to demonstrate the best surgical approach for ERP. The authors observed an improvement in Hologram baseline, but no differences were found among the randomized comparisons. Following the PROSPER study, there has been increased use of minimally invasive AA, with a corresponding reduction in PA^28^.

A study of 50 patients with ERP were randomized into either ventral rectopexy or Delorme procedure, with recurrence rates of 8 per cent and 16 per cent, respectively^6^. The results of the current meta-analysis are in keeping with the literature, with a recurrence rate of 9.8 per cent for the AA and 16.3 per cent for the PA. These data were consistently found in all included studies, apart from three^7,22,26^. In the study by Lee *et al*.^22^, the small sample size of AA (eight patients) *versus* PA (123 patients) might explain this finding, in contrast to the low number of PAs included in the study by Sobrado *et al.*^26^, which demonstrated a higher risk of recurrence after AA. Interestingly, the current meta-analysis found that recurrence rates for AA were higher in RCTs *versus* non-RCTs (9.8 per cent *versus* 7.7 per cent), whereas for PA they were lower in RCTs *versus* non-RCTs (16.3 per cent *versus* 20.1 per cent), which resulted in the overall difference not being statistically significant in the meta-analysis of RCTs. Even if this difference might still be clinically relevant, it warrants further prospective, long-term evaluation.

The current meta-analysis demonstrated that age is an important factor in predicting recurrence, with both approaches having higher recurrence rates in patients older than 65 years. Lieberth *et al.*^29^ reported an overall recurrence rate of 14 per cent in 70 patients operated on using the Delorme technique, but this decreased to 8 per cent when only patients under 50 years of age were considered. Fu *et al.*^30^ reported a recurrence rate of 22.1 per cent from a population of 113 patients who underwent laparoscopic ventral rectopexy, reporting age over 70 years as a predictive factor (hazard ratio 2.22). In contrast, a more recent consensus statement published by the Italian Society of Colorectal Surgery^2^ suggested that age should not be considered as a determining factor in the choice of treatment, as even in older patients the two procedures would be similar.

Patients having a PA with a mean duration of follow-up of more than 40 months had a higher recurrence rate. Conversely patients having an AA with a mean duration of follow-up of less than 40 months had higher recurrence rates. This might suggest that recurrence in the AA may occur earlier than in PA.

Few studies have examined the impact of these procedures on HRQoL and clinical symptoms, but both approaches are effective in achieving improvement in symptoms associated with ERP. A proportion of patients who undergo surgical repair continue to present with symptoms such as obstructed defaecation, constipation, and incontinence. Some studies have shown that the persistence of obstructive defecation, its exacerbation, or even its *de novo* occurrence is more common with AA than with PA^31^. Constipation may also occur after abdominal rectopexy, with a reported incidence ranging from 27 to 47 per cent. The reason for this phenomenon is unknown, although it has been hypothesized that it could be due to scar around the rectum and resultant stiffness^32^.

PA may be associated with an increased frequency of bowel movements, urinary urgency, and faecal incontinence, with the incidence reaching 40 per cent^33^. This could be explained by the reduced capacity and compliance of the rectal wall. Constipation during perineal surgery is reported in about 10 per cent of cases^34^. The current study showed that the risk of incontinence was three times higher in PA than in AA; in contrast, the risk of constipation might be higher in AA procedures. However, this was not demonstrated when analysing RCTs separately. The risk of constipation was still higher with PA than with AA in this subgroup of studies (*Fig. 4*), which merits further investigation.

Given the reported data, the choice of procedure to perform in each patient should be individualized. The relatively high number of recurrences after PA is potentially balanced by the less invasive technique and by the option of redo procedures^35^.

It is important to consider the complication profile of different approaches, for example damage of hypogastric nerves with an AA when mobilizing the rectum. This can cause subsequent bowel, bladder, and sexual dysfunction. Unfortunately, no consistent objective data that can be compared are available on this topic, which might be relevant to guide the choice of the type of approach.

Duration of stay was addressed in the current meta-analysis because this might be of interest to patients and hospital management. Using techniques such as PA or minimally invasive AA to reduce hospital stay to a minimum can be useful when limited resources and staff are available^36,37^.

This review has limitations, mainly consisting of the limited number of RCTs available and the low quality of the studies. These considerations warrant careful evaluation and the results need to be interpreted with caution. The heterogeneity of available data limited the possibility of sensitivity and subgroup analyses. There is increased adoption of minimally invasive surgery, which might have an influence on the outcomes.

However, this study has identified areas for further research, including HRQoL and functional outcomes. Several factors relevant when counselling patients on the different treatment approaches to ERP were identified, and decisions should be made within the context of the multidisciplinary team. Follow-up for functional diseases should be long enough to ensure that all recurrences are detected, ideally assessing both anatomical and functional outcomes^38^. Future studies on ERP are needed, but these do not necessarily need a randomized design. Properly executed non-RCTs might provide useful real-world data and could be more cost-effective. These studies are needed to clarify how surgery impacts on function and HRQoL, using validated tools and incorporating patient-reported outcome measures and patient priorities.

## Supplementary Material

zrac018_Supplementary_Data

## Data Availability

Additional data can be found as supplementary material and are available from the corresponding author on reasonable request.
