# Supplementary material for: Abdominal versus perineal approach for external rectal prolapse: systematic review with meta-analysis
Source: BJS Open. 2022 Apr 7;6(2):zrac018. doi: 10.1093/bjsopen/zrac018 (PMC8989040; doi:10.1093/bjsopen/zrac018)
Supplement: zrac018_Supplementary_Data [file zrac018_supplementary_data.zip › Supplementary_Table_1.docx]

**Table S1.** Detailed search strategy

| **Database** | **Time span** | **Search strategy** |
| --- | --- | --- |
| MEDLINE (PubMed) | January 1990 to December 2021 | ((((((Delorme) OR Altemeier) OR Perineal Approach) OR Abdominal approach) OR Rectopexy) AND Rectal prolapse) AND ("1990/01/01"[Publication Date] : "2021/12/31"[Publication Date]) |
| Embase | 1990 to 2021 | (Delorme OR Altemeier OR (abdominal AND approach) OR (perineal AND approach)) AND rectal AND prolapse |
