# Supplementary figures and images for: Abdominal versus perineal approach for external rectal prolapse: systematic review with meta-analysis
Source: BJS Open. 2022 Apr 7;6(2):zrac018. doi: 10.1093/bjsopen/zrac018 (PMC8989040; doi:10.1093/bjsopen/zrac018)

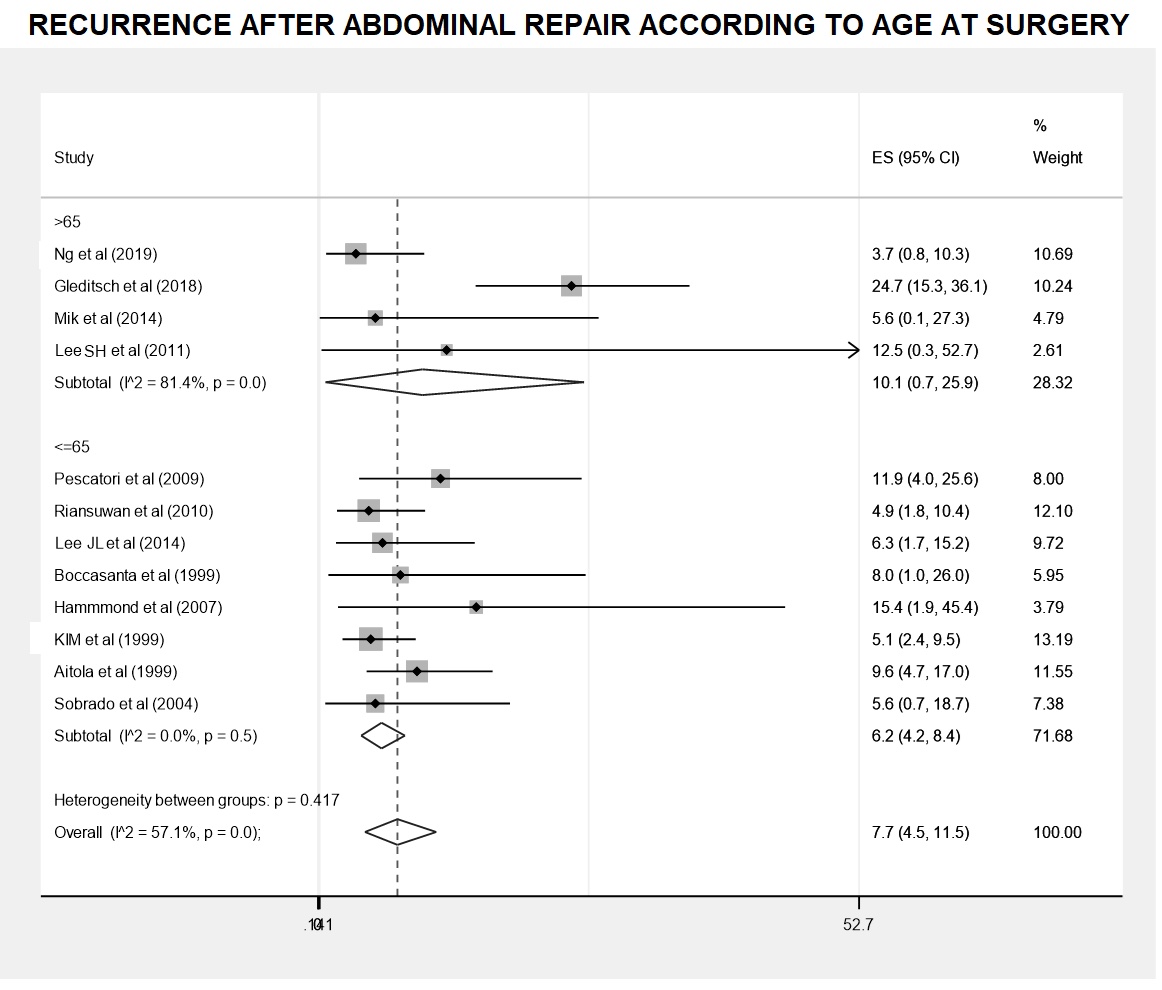

Supplement: zrac018_Supplementary_Data [file zrac018_supplementary_data.zip › Supplementary_Figure_1.jpg]

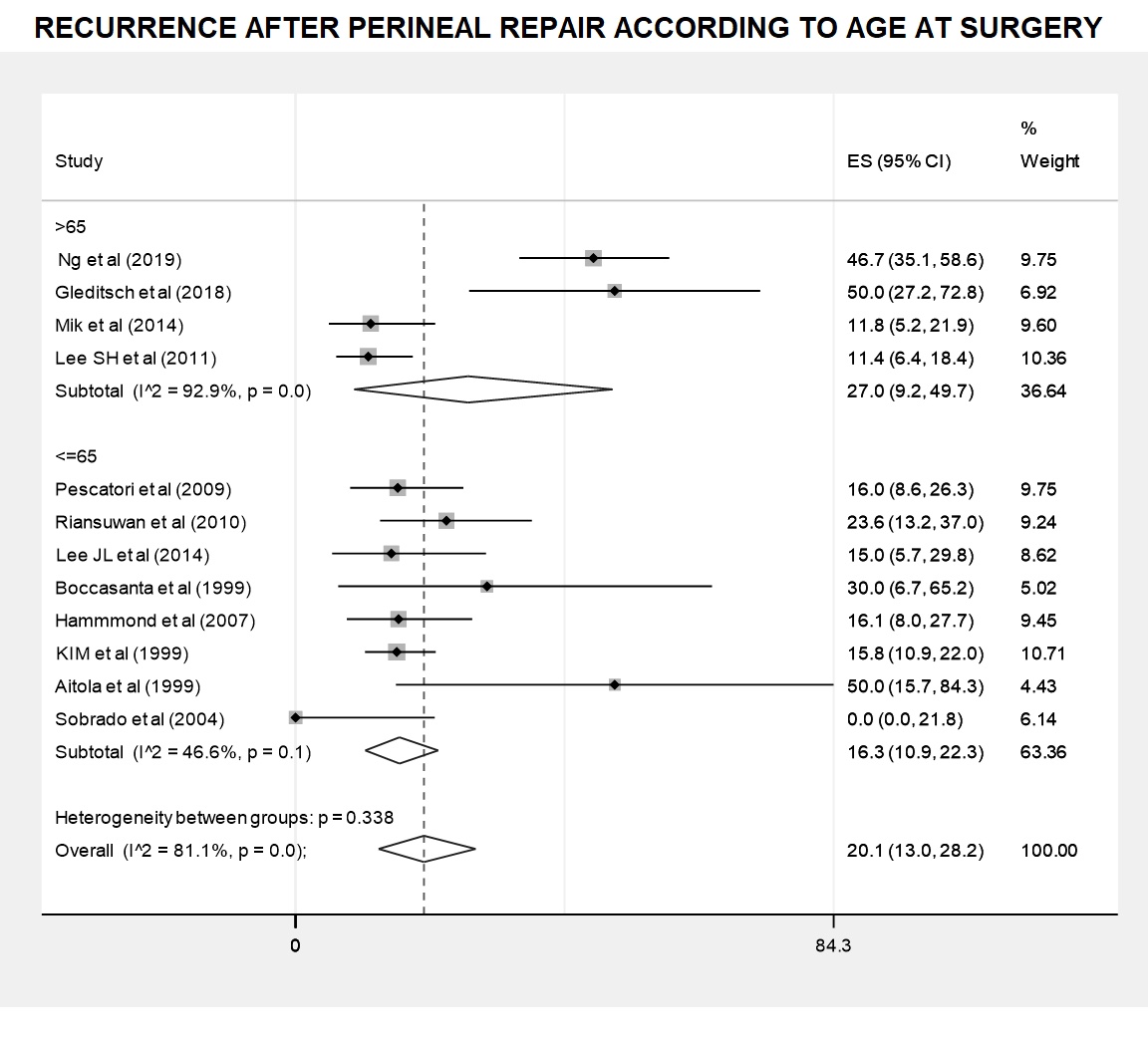

Supplement: zrac018_Supplementary_Data [file zrac018_supplementary_data.zip › Supplementary_Figure_2.jpg]

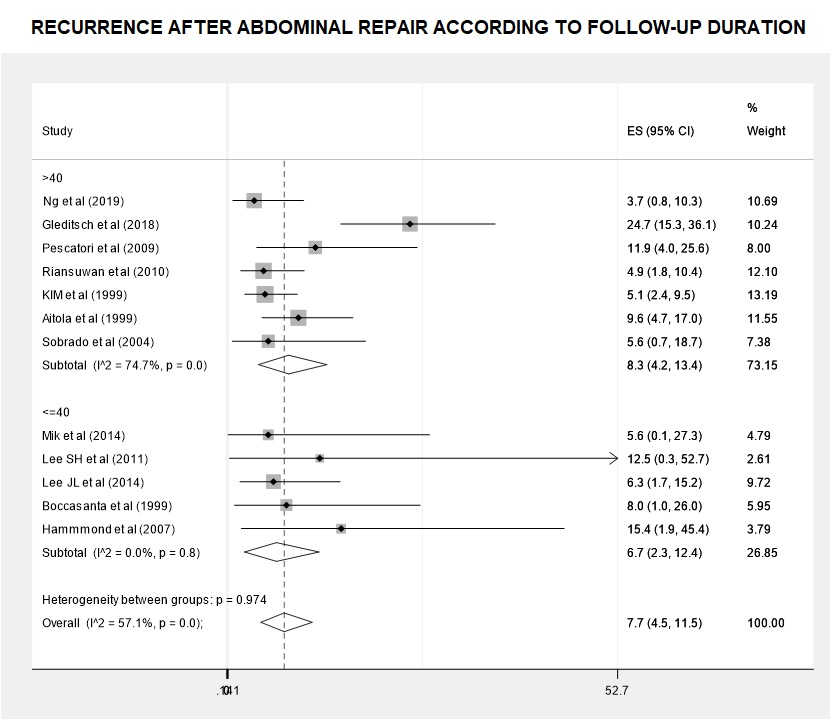

Supplement: zrac018_Supplementary_Data [file zrac018_supplementary_data.zip › Supplementary_Figure_3.jpg]

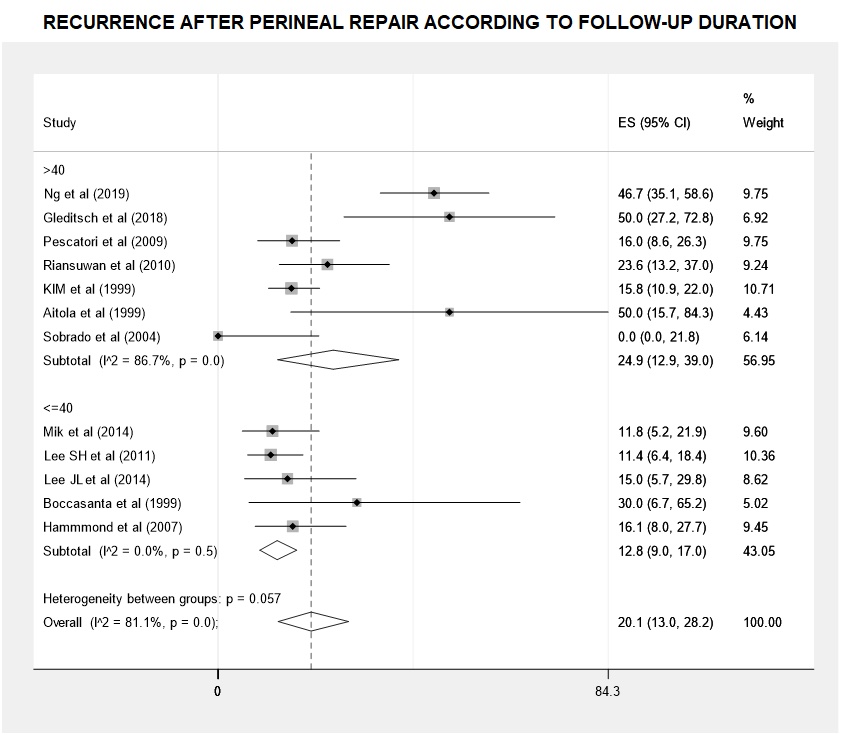

Supplement: zrac018_Supplementary_Data [file zrac018_supplementary_data.zip › Supplementary_Figure_4.jpg]

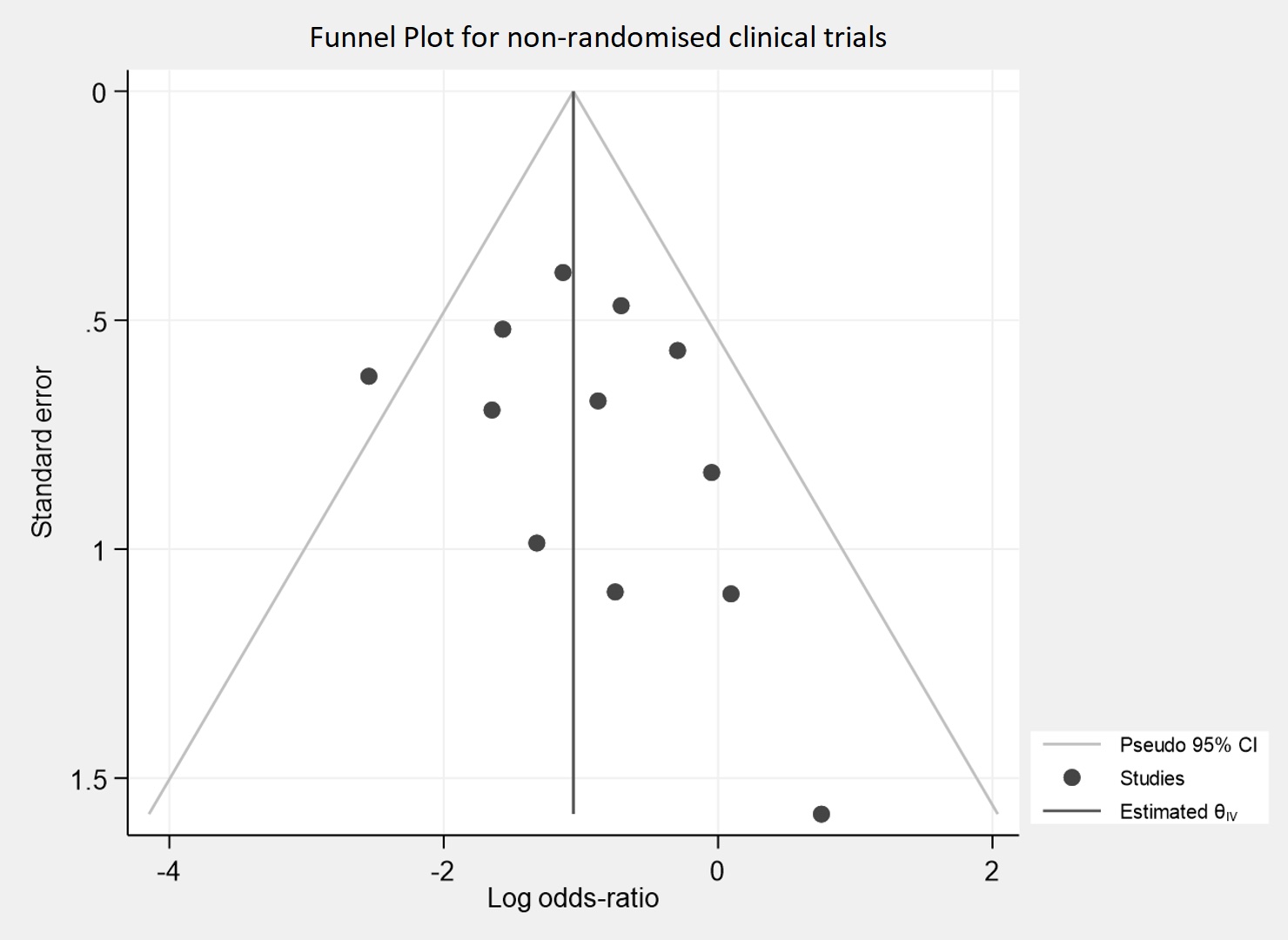

Supplement: zrac018_Supplementary_Data [file zrac018_supplementary_data.zip › Supplementary_Figure_5.jpg]
